# Dual antiplatelet therapy in GI-bleed patients with extensive coronary artery disease history: a systematic review

**DOI:** 10.1097/MS9.0000000000002833

**Published:** 2025-04-25

**Authors:** Abdulrashid Onimisi Abdulrahim, Mohannad Jawad Yahya Abd-Alhadi, Hussein Attia Hussein Mahmoud, Ayesha Sarwar, Anum Akram, Walter Jauregui Alvarado, Olamide Sadik, Srija Reddy Kesireddy, Hassan Mumtaz

**Affiliations:** a Gastroenterology Specialist. Fellowship NPMCN, King Fahd Specialist Hospital Buraydah, Al Qassim province, Saudi Arabia; b AbdulHadi Hospital, Amman, Jordan; c Diagnostic Radiology Fellowship Heliopolis Hospital, Cairo, Egypt; d Dr. V.R.K. Women’s Medical College, Hyderabad, India; e Jinnah Sindh Medical University Karachi, Pakistan; f Universidad Nacional Autónoma de Honduras, Tegucigalpa, Honduras; g Richmond Gabriel University, St. Vincent and the Grenadines; h SVS Medical College, Mahabubnagar, India; i BPP University, London, United Kingdom

**Keywords:** antiplatelet therapy, coronary artery disease, gastrointestinal bleed, GI bleed

## Abstract

**Introduction:**

Extensive coronary artery disease whether at initial presentation or after percutaneous coronary intervention (PCI), involves the use of dual antiplatelet therapy (DAPT) as a very significant therapeutic option. There are many reasons why clinicians should weigh the risks of bleeding and cardiovascular thrombosis when deciding whether to maintain or discontinue DAPT in such patients.

**Methods:**

This comprehensive review systematically analyzed via electronic databases, a total of 39 papers most of which were on countries in Southeast Asia. The focus was on randomized control designs (RCTs) and observational studies.

**Results and discussion:**

PCI with drug eluting stents was the most common method of treating acute coronary syndrome. The study found more independent predictors of gastrointestinal (GI) bleeding in young and elderly patients from Southeast Asia. The precise DAPT score was more readily used among various bleeding risk prediction models for patients on antiplatelet therapy.

**Conclusion:**

The use of DAPT in the setting of the GI bleeding risk in patients with coronary artery disease has been studied extensively, but there are still no clearly defined strategies and very definite answers to the risk of bleeding versus cardiac event envisaged during the management of an extensive acute coronary disease mostly in southeast Asia. More studies using good study designs and statistics and establishing clinical prediction rules, are needed to fill this knowledge gap most especially in Southeast Asia and Middle East.

## Introduction

The initial treatment of coronary artery disease involves antiplatelet medication, especially after percutaneous coronary intervention (PCI). Physicians are aware that serious gastrointestinal (GI) bleeding is a typical consequence of these drugs in addition to other hemorrhagic problems. Because of this, it is critical to identify risk factors for bleeding complications in patients undergoing dual antiplatelet therapy (DAPT)^[^1^]^ such as being over the age of 75, being a smoker or alcoholic, having anemia, cancer, peripheral artery disease (PAD) or coronary artery disease (CAD), liver or renal dysfunction, a history of stroke, or any other bleeding complications, and so on^[^2^]^. Risk factors may be more quickly identified with the practice of regular clinical examinations of such patients^[^3^]^.

Various tools have been formulated to identify hemorrhagic risk prediction in patients with cardiovascular disease (CVD) on DAPT. These tools vary in their methodology and origin. Their effectiveness, however, is moderate. These tools aid in effective decision making regarding the risks versus benefits of the dual antiplatelet therapies (DAPTs). Besides, it assists in keeping the patient well educated about the possible risks of bleeding and thereby, a vigilant treatment plan can be formulated as per individual patients^[^4^]^. While considering age more than 65 years, recent bleeding, proton pump inhibitor (PPI) usage, dual anti-platelet therapy (DAPT) usage, and the usage of combined anticoagulants, a new risk scoring model have been formulated to assess the risk of an acute GI-bleed. Other scores include CRUSADE and S2TOP-BLEED score which are used to predict intra-ocular bleeding, GI bleeding and Intracranial hemorrhage. The (ABC)2D score was found to be more effective than CRUSADE score^[^5^]^.

By considering Japanese PCI studies, a score which assesses both thrombotic and bleeding risks called CREDO-Kyoto, was formulated with moderate accuracy^[^6^]^. The idea of individualized anti-platelet therapy gained more significance when different platelet reactivities have been observed on P2Y12 inhibitors, such as high and low platelet reactivity, which predicts ischemic and bleeding risks^[^7^]^.

Ethnic differences in GI-bleed are also found in several studies as a formerly known concept is the “East Asian Paradox,” meaning, the occurrence of cardiovascular events (like ischemia) is higher in western countries than Asia, whereas bleeding adverse events were higher in Asians. This difference can be attributed to the presence of a poor metabolizer CYp2C19 in Asians^[^8^]^.

In today’s practice, the DAPT regimen of either 1 or 3 months followed by single antiplatelet therapy is found to have low bleeding risk without increasing the ischemic risks of stent thrombosis. There is still a breach in knowledge as to how to decide on 1- or 3-month DAPT regimen and the significance of single anti-platelet therapy after DAPT use. Long term use of DAPT is not recommended as interrupting the use of the drugs due to noncompliance for bleeding can lead to more adverse events^[^9^]^.

It is worth mentioning that a few cost-effective analyses on durations of DAPT after PCI showed that short term (i.e., 3–4 months) of DAPT is better than the ≥12-month period. Thus, judicious usage of DAPT regimen is very much essential to reduce this global burden^[^10^]^.

Prevention of GI-bleed in patients using DAPT is mainly done by PPIs which are predominantly used for acid-related disorders, like, gastroesophageal reflux disease (GERD)^[^11^]^. This prevention has still not been very well established due to knowledge gaps, difficulties in decision making and professional role of PPIs. Multicompetent intervention would be required to formulate guidelines for using PPIs to prevent GI-bleed^[^12^]^.

Moreover, additional light needs to be shed on topics like identification of high-risk individuals for bleeding, how to reduce non-upper-GI bleed risks, finding equilibrium between risks and benefits in geriatric population in both primary and secondary prevention^[^13^]^.

Different views and clinical approaches in handling anticoagulants and antiplatelets are noticeable among endoscopists from the east and the west. The absence of consistent practices emphasizes the need for more research and better education in the field of GI endoscopy for patients on anti-platelets and anti-coagulants, especially considering the impact of individual patient characteristics on specific complications^[^14^]^.

Additional the study is called for, to correlate high risk factors of hemorrhagic complications noticed in patients from East Asia as opposed to the patients from other parts of the world. Moreover, a study of the causes of bleeding manifestations in certain clinical situations, in East Asians, is required^[^15^]^.

## Methodology

This systematic review was conducted following the preferred reporting items for systematic reviews and meta-analysis (PRISMA) guidelines assessed in January 2024. Our work has been reported in line with AMSTAR (Assessing the methodological quality of systematic reviews) Guidelines.

### Eligibility criteria

We considered published studies within a period of approximately last 20 years including prospective observational studies (cohort studies), retrospective observational studies (both cohort and case control studies) conducted in any adult population, randomized-single/double blind control trials, studies on randomized control trial (RCT) reviews that included clearly stated end points, post hoc analysis of ongoing clinical trials-in phase 3 and those that reported measures of association for example odds ratio, hazard ratio, risk ratio with their corresponding 95% confidence intervals.

### Exclusion criteria

Studies that were excluded were letters, editorials, duplicate studies as noted on the Google sheets, narrative reviews, study protocols and guidelines and systematic reviews with no clearly stated end points on a meta-analysis.

### Literature search

Search strategies – Detailed literature search was conducted using electronic data bases PubMed/Medline, Web of Science, Google scholar, Embase and Scopus. full texts were assessed using the Elsevier, Wiley, Springer journal suggester, Oxford academia, PMC, BJCP, and BMJ free article assess links, NIH public access and IEEE publication reminders. Manuscript matcher accessed via end note were also used on the first 73 papers searched for.

Papers published between 2004 and 2024 were identified, if it pertained to DAPT and/or GI bleed patients with coronary artery history.

The key words – Medical subheading (MESH) terms utilized for the database searches. They were pertinent clinical terms that are synonymous with “acute coronary syndrome (ACS),” “percutaneous coronary intervention (PCI),” “coronary artery disease (CAD),” “cardiovascular disease (CVD),” and “antiplatelet therapy” in “gastrointestinal bleeding (GIB).” Studies published in English language were the only ones evaluated, with translated studies also being considered. Articles focusing on the Southeast Asian area were given precedence.

### Screening and data extraction

Screening eligible studies and data extraction using a Google sheet were performed independently by two group coordinating steering committee made up of three research authors in each group. The full texts were made available when there were difficulties by the main research coordinator. Group A – names (OI, WJ, & An), Group B – names (AA, Hu, & MA). Two researchers (SVR & AS) used the available research materials during the screening process to conclude an introductory page as a form of pre-evaluation pattern and suggesting the precise end points needed in the review. This was based on initial extracted reports we found in the literature search.

### Data extracted from each eligible study included

End points in the form of major findings and covariates – if necessary, first authors’ surnames, year of publication, country of study, study title, name of journal, study design, sample size, duration of follow-up, patient characteristics and treatment procedures, statistical approach, and so on.

## Results

In the initial search that yielded 73 literatures on Google sheets number (GSN) 3–75. Non duplicate records, were – 68 in number of which 14 more were excluded for reasons mainly due to lack of full access to the article and due to lack of relevance to the subject title of the paper. Please see Google sheets attached file.

In articles 3–75, there were 27 articles that satisfied our inclusion criteria (eligibility criteria). There were two post hoc analyses of ongoing RCT (GSN 33 & 50) and three RCT studies (GSN 5, 26, & 75). Two case control studies (GSN 8, 11), six prospective observational cohort studies (GSN 4, 10, 12, 42, 46, and 52). Nine systematic analyses of multiple RCTs (GSN 27, 35, 36, 38, 39, 60, 65, 68, and 74); and five retrospective cohort studies (GSN 29, 40, 41, 44, and 55).

Details of excluded papers from the results analysis in this review, were three guidelines or expert consensus – (GSN) 54, 57, & 63 and 22 review articles (8 of which had to be excluded in discussion of results due to lack of clear relevant statistical analysis or lack of clear end point of interest). Eight studies were not summarized as there were conflict of interests – these were (GSN) 17–25.

**Figure 1.**
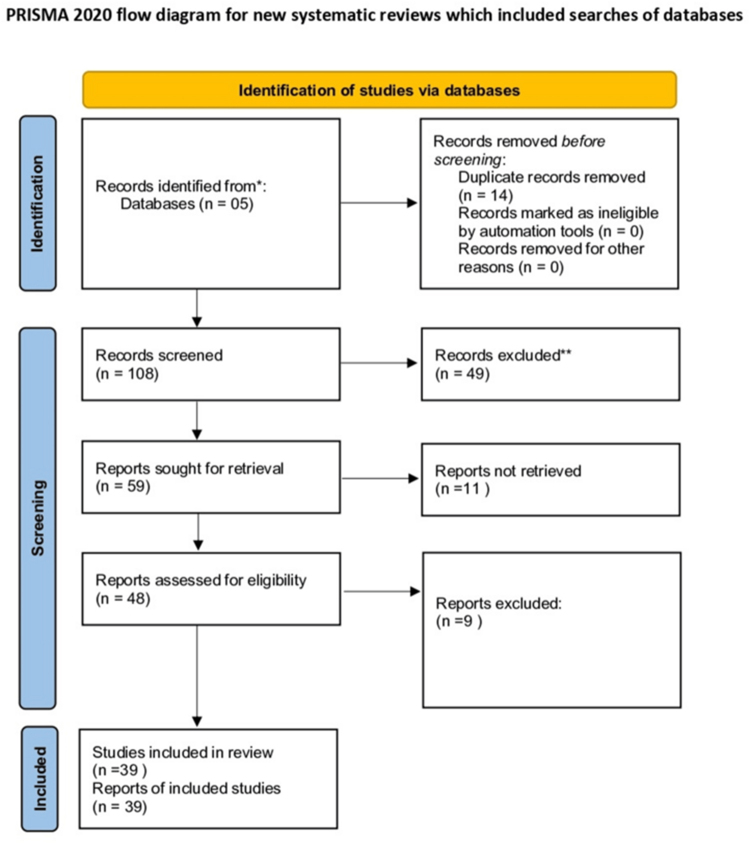
PRISMA 2020 flow diagram for new systematic reviews which included searches of databases.

A second search of literature was done during discussion of results and a further 12 eligible studies were extracted which included three RCT – (GSN) 79, 90, & 93; one prospective cohort – (GSN) 86: four retrospective cohort studies – (GSN) 77, 89, 90, & 98: one case control study – (GSN) 97 and three systematic review on RCTs – (GSN) 78, 82, 83, as shown in Fig. 1.

### Evaluation “quality checks” of included studies and summary of the studies characteristics

Research from different nations is shown in Table 1 of the systematic review data. These countries included Israel, Korea, Thailand, China, Japan, Saudi Arabia, Canada, Russia, Spain, Italy, the United States, and Switzerland, among others. Research methods ranged from meta-analyses to systematic reviews, retrospective cohort studies, prospective observational studies, and multicenter randomized controlled trials (RCTs). The studies had varying follow-up lengths, a few were performed as post hoc analysis of longer-term trials see Vaduganathan *et al* gsn 33 & Nikolsky *et al* gsn 52, others had a constant follow-up duration of 30 days to 15 months which complies with the nature of the trials and one paper with a single RCT mentioned some conflicts of interests – see Chan F K L *et al* gsn 75. Statistical analysis recognized on all the included papers in Table 1, were made in respect to the stated objectives of the research conducted and endpoint of the trials were acceptable.

**Table 1:** Research from different nations of the systematic review.

GSN & Study first author year & country	Title	Journal	Study design	Follow up	Baseline characteristics	Type of ACS management	Main study outcome	Statistical analysis	Adjusted HR/OR (95% CI), *P* value, other	Additional drug management
4. Thanapirom *et al* 2011 Thailand	Outcome of acute upper gastrointestinal bleeding in patients with coronary artery disease	Saudi Journal of Gastroenterology 2016	Prospective multi-center cohort study	Median follow up unavailable. Study period – 21 months	Coronary artery disease patients (CAD)	Conservative management & Others-revascularization therapy	Risk factors; Upper gastrointestinal bleed (UGIB) patients with CAD-older, antiplatelet and warfarin vs non-CAD cohort.	Matched case-control analysis done with two sample *t* tests and multivariate logistic regression analysis.	CAD vs non-CAD cohort-Higher UGIB scores, i.e., Glasgow Blatchford, Rock all Scores, but similar outcome – mortality rate, rebleeding, surgery, embolization & packet erythrocyte transfusion.	Antiplatelet drugs – not specified. Anticoagulant-warfarin
5. Chung C S *et al* 2022 Taiwan, Japan	Randomized controlled trial of early endoscopy for upper gastrointestinal bleeding in acute coronary syndrome patients	Scientific Reports 2022	Multicenter randomized controlled trial	Variable per patient. Terminated study due to slow enrolment	ACS ± DAPT with UGIB & Endoscopy done	Revascularization strategies PCI and/CABG. variable – full details unavailable	Early endoscopy (EE) vs non-early endoscopy (non-EE) group-better outcomes of Failure to control hemorrhage – 4.55% vs 23.81%. *P* < 0.001. 3-day rebleeding rate – 4.55% vs 28.57%. *P* = 0.033	Intention to treat (ITT) & Per-protocol (PP) analysis done. Chi-square, Fisher exact, univariate & multivariate logistic regression analysis.	Discontinued DAPT patients during acute UGIB – risk higher for coronary stent re-thrombosis within 6 months = OR – 5.25 (1.21–22.74). Others-male sex. After EE – OR = 3.50 (1.55–10.63) for minor complications & 4.25 (1.43–12.63) for major complication rates.	Antiplatelet therapy – different combinations, proton pump inhibitors – different types. Blood transfusion.
8. Alli O *et al* 2007 USA	Incidence, predictors, and outcomes of gastrointestinal bleeding in patients on dual antiplatelet therapy with aspirin and clopidogrel	Journal of clinical gastroenterology	Observational, case-control study	4 years	CAD on DAPT	PCI (Drug eluting stents-DES) ± CABG	The rate of GI bleeding was 2.7%. Gastritis and gastric ulcers most common findings in 49% at endoscopy. History of GI bleeding is most important independent predictor of GI bleed. *P* < 0.001	*t*-test, Fisher exact test. Multivariate logistic regression analysis	30-day mortality rate – in GI bleeding & control groups – 3.7% & 0% respectively. Corresponding 1 year mortality rate – in GI bleeding & control groups – 3.7% and 0% respectively. *P* < 0.01	Aspirin, Clopidogrel. Aspirin + Clopidogrel/other antiplatelet agents.
10. Nagata N *et al* 2014 Japan	Lower GI bleeding risk of nonsteroidal anti-inflammatory drugs and antiplatelet drug use alone and the effect of combined therapy.	Gastrointestinal Endoscopy Journal 2014	Prospective (single center) cohort study	Not available	Lower GI bleeding patients -LGIB (acute continuous, or frequent) on NSAID or antiplatelet agents.	Not stated	The single use of non-selective NSAID or cyclooxygenase − 2 inhibitor was independently associated with LGIB.	Univariate and multivariate regression analysis.	Adjusted HR/OR (95% C.I.), *P* value, other.	Additional drug management
11. Ibanez L *et al* 2005 Spain	Upper gastrointestinal bleeding associated with antiplatelet drugs	Alimentary Pharmacology & Therapeutics 2006	Case-control study	10 months	Patients older than 18 yrs. ago with cardiovascular disease & others requiring antiplatelet agents	Not available	Individual risks of UGIB were cardiovascular-Concomitant PPI reduced all risk estimates. Antiplatelet drugs accounted for 14.5% of all cases of UGIB for >70 yrs old	Univariate and multivariate regression analysis	The combined use of NSAIDs with low-dose aspirin (OR 4.3) or with other antiplatelets (OR 4.9) was more associated with LGIB than the use of NSAIDs alone (OR 2.3)	NSAIDS Antiplatelet agents-Among is Aspirin, clopidogrel, ticlopidine used as stated.
12. Sostres C *et al* 2019 Spain	Risk of rebleeding, vascular events and death after gastrointestinal bleeding in anticoagulant and/or antiplatelet users	Alimentary Pharmacology & Therapeutics .2019	Observational cohort study	24.9 months	All disease (including CVD) requiring anticoagulant or antiplatelet therapy	Not available	Resumption of both anticoagulant or antiplatelet therapy associated with higher risk of rebleeding & lower risk of ischemic events or death: similar for UGIB & LGIB	Univariate analysis	-Cardiovascular drug risk factor Aspirin-OR = 4.0 (3.2–4.9)	
									Clopidogrel -OR = 2.3 (0.9–6.0)	
									Dipyridamole -OR = 0.9 (0.4–2.0)	
									Ticodipine-OR = 3.1 (1.8–5.1)	
									Triflusinal-OR = 1.6 (0.9–2.7)	Antiplatelet drugs as stated, Ibuprofen.
26. Sunil v Rao *et al* 2005. USA	Impact of bleeding severity among patients with ACS	American Journal of Cardiology. 2005	4 Multicenter RCT	6 months	ACS ± CABG	CABG	27.6% Prevalence of ≥ 1 bleeding episode. Same consistent ± CABG	Cox proportional hazards 30 days/6 months for stepwise	Rebleeding rates higher in anticoagulant vs antiplatelet (138.0 vs 99.0 events per 1000 patient years).	
								Mild-		
								Moderate-	Bleeding location identical in 16.8% of cases.	
								Severe-bleeds respectively		
27. G. Ter Salvi *et al* 2020. Switzerland	ACS, antiplatelets therapy & bleeding. Clinical perspective	Journal of Clinical Medicine 2020	6 RCTs	3 months to 15 months (max trial)	ACS on DAPT	PCI-only	Reduction of bleeding events & worsening ischemic outcomes for PY2Y12 inhibitors.	Meta-analysis	Adjusted HR/OR (95% CI), *P* value, other.	Additional drug management
29. A Shalev *et al* 2012. Israel	Incidence, predictors and outcomes of UGIB in patients with ACS.	International Journal of Cardiology 2012	Retrospective cohort for 11 years	30 days end point	ACS on revascularization strategy	Multiple	Prevalence – 89% vs 68% (*P* = 0.03)	*T* tests,	HR – 1.6–10.6.	ASA
							30-day mortality – 33% vs 5%, *P* < 0.001	Mann–Whitney *U* tests &	(1.3 up to 13.6) stepwise increase	DAPT &
								Correlation analysis	HR – 1.4–7.5 (1.2–9.3) stepwise.	PPI
33. Vaduganathan *et al* 2015 Intercontinental-global	PPI’s reduce gastrointestinal events regardless of aspirin dose in patients requiring DAPT	Journal of American College of Cardiologist 2016	RCT-post-hoc of COGENT trial lasted 1 yr.	110 days (median)	ACS only-on PPI vs placebo	Multiple revascularization strategies	High dose aspirin -similar to low dose estimates of bleeding events.	Chi-square, fishers exact, *t* tests and Kaplan–Meier estimates at 180 days	Not available	Clopidogrel
										Ticagrelor
										Prasugrel
										Tailored therapy…
										PY2Y12 inhibitors
							PPI reduction of 180-day hazard.	Cox proportional hazards models.		
35. Khan M Y *et al* 2018 USA	Reduction in post percutaneous coronary intervention angina	European Journal of Gastroenterology. Hepatology 2018	5RCTs	Variations	ACS and PPI ± DAPT and	Multiple revascularization strategies	Concomitant	Rev-Man, Version 5.3	Heparin & glycoprotein IIb/IIIa-UGIB higher risk: OR – 2.87 (1.66–4.97)	PPI
			Other cohort and case control studies.				PPI with P2Y12 inhibitor protective against GI events			Unfractionated heparin
										GP IIb/IIIa inhibitors
36. Zhu Y *et al* 2022 China	PPI in the prevention of mucosal injury associated with DAPT after CABG. DACAB-GI-2 study	Trials 2022	RCT	12 months	ACS on DAPT	PCI with stents (unspecified), CABG.	Good efficacy & Safety of both 12 months & 1 month PPI-pantoprazole prophylaxis	Cox proportional model	Adjusted HR/OR (95% C.I) *P* value, other.	Additional drug Management
38. Al mufleh A *et al*. 2016 Canada & Saudi Arabia	H2 Receptor antagonists vs PPIs in patients on DAPTs for CAD.	Cardiology 2018	10 RCT Evidence based review-Cochrane central register of controlled trials.	Variable	Coronary artery disease on DAPT	Multiple revascularization strategies	PPIs associated with HTPR and less MACE implications	Fixed effects meta-analysis	GI events – 1.7% vs 2.1%. aHR – 0.88 (0.46–1.66) MACE 4.8–5.5%. aHR – 0.73 (0.48–1.11)	Various PPI’s Low dose Aspirin. High dose Aspirin, Clopidogrel, Other antiplatelet agents, Oral anticoagulant agents.
39. Tanigawa T *et al* 2011 Japan	GI bleeding after PCI	Digestion 2011	Evidence based review -RCT, case reports, case series, case control and cohort studies	Variable	ACS patients after PCI	PCI ± DAPT	Prevalence of GI bleed of 2%	Sample *T* tests, Fischer exact and chi-square tests. Logistic regression analysis.	GI bleed reduction – 22 vs 66.	Various PPI’s
										Aspirin,
										P2Y12 inhibitors
									OR – 0.37 (0.23–0.61)	
									*P* ≤ 0.001	
									Inc of unstable Angina-46 vs 67.	
									OR – 0.67 (0.45–0.99)	
									*P* = 0.005	
40. Huang K W *et al*. 2012 Taiwan	Risk factors of UGIB in CAD patients receiving both aspirin & clopidogrel.	Journal of China Medical Association 2013	Retrospective cohort study (1 yr.)	Mean follow up − 125 days)	ACS on DAPT ± PCI	PCI (elective, primary or urgent)	Similar early and late stages GI bleed complication. Protective role of PPI.	Cox regression analysis	Reduced GI bleed events	Clopidogrel + Aspirin vs Ticagrelor + Aspirin
41. Yasuda H *et al*. 2009 Japan	UGIB in patients receiving DAPT after coronary stenting	Internal Medicine 2009	Retrospective cohort study (2 yrs.)	2 yrs.	Coronary heart disease after revascularization treatment	CAG & previous PCI (DES)	Cumulative incidence – 1 & 2 yr (lack of antisecretory therapy) – 4.5 & 9.2%	Logistic regression. Kaplan-Meier estimates	Reduced GI complications-OR – 1.28 (0.17–0.48)	Additional drug management
									HTPR-OR – 1.28 (1.030–1.60)	
									MACE-OR – 0.99 (0.55–1.77)	
42. Komarov A L *et al* 2021 Russia	Gastric mucosa condition in patients with coronary artery disease and high risk of gastrointestinal bleeding (register REGATTA)	Therapeutic Archive 2022	Single center prospective study	1–15 months post GI bleed.	Long term antithrombotic therapy patients.	PCI in only half of population.	Incidence & Sources of GI bleed endoscopically – 2%/yr., multiple respectively.	Mann-Whitney, Fisher exact tests (two sided)	GI bleed after PCI risk factors associated with increased morbidity, mortality, duration of hospital stay & cost	PPI’s (various)
										H2RA’s
				Median – 12 months	Stable coronary artery disease.					
							Failure of prevention of large/multiple erosions or ulcers by PPI therapy.			
43. U Idiazabal *et al* 2021 Spain	Differences of GI bleeding after PCI (+Stent) among different DAPT.	European Heart Journal 2021	Retrospective observational cohort study	5 yrs.	ACS on PCI	PCI (+Stent)	GIB events.	Multivariate logistic regression	PPI protective: HR – 0.10 (0.01–0.71)	PPIs
							MACE			
									New ACS: HR – 2.67 (1.33–5.34)	H2RA’s
46. Arroyo R C *et al*. 2012 Spain	Lower GI bleeding is more common than upper among patients on DAPT.	Heart 2012	Prospective observational study	1.72 ± 1.07 yr. per patient	ACS on PCI ± DAPT & PPI	PCI only	Frequency of lower GI bleed – 1.52/100 patients.	Multivariate logistic regression analysis.	Adjusted HR/OR (95% CI), *P* value, other.	Aspirin
										Clopidogrel
										PPI
							Mean time to bleeding event – 1st year = 7.03 ± 7.65mths. (84.6% patients on long term PPI at time of bleed)			
50. Koskinas K C *et al*. 2009–2012 Switzerland	Clinical impact of Gastrointestinal bleeding in patients undergoing PCI.	Circulatory Cardiovascular Interventions 2015	Prospective observational study	1 yr.	ACS on PCI	PCI + (DES -different, Bare metal stent)	Triple antithrombotic therapy-single drug related predictor of Gastrointestinal bleeding & some patient related factors.	Multivariate logistic regression analysis.	Protective effect of antisecretory therapy significant. *P* = 0.044	Additional drug management
						Balloon angioplasty				
									PPI Rx grp-More adverse cardiac event-significant. *P* < 0.05	
52. Nikolsky *et al*. 2005 Multiple countries.	Gastrointestinal bleeding in patients with ACS: Incidence, predictors, & clinical implications	Journal of American College of Cardiology 2009	RCT – post-hoc analysis	30 months	ACS on single/multiple revascularization strategy.	PCI (DET, BMT, both)	GIB strongly associated with mortality and composite ischemic end point.	Multivariate logistic regression analysis.	Failed PPI prophylaxis – 18.2%	PPIs
						CABG				
								Kaplan-Meier estimates	H. pylori – 90.9% of patients.	H2RA’s
55. L. Zhong *et al* 2018 China	Risk factors for Gastrointestinal Injuries in ACS Patients with DAPT in 1 yr. Follow up	World Journal of Cardiovascular Diseases 2018	Retrospective cohort study (Jan. 2014–Aug. 2015)	1 yr.	ACS on DAPT	Not available	1 yr. frequency of symptomatic GI injury – 17.9%.		No differences in incidence of GI bleed among – 3 optimized DAPT: Ticagrelor grp-OR = 1.293. *P* = 0.154	
							-Peaked in first 3 months.			
							Independent predictors of serious GI complications – Drinking habit & previous peptic injury			
									Prasugrel grp-OR = 0.667. *P* = 0.111.	
								Cox Regression analysis	Less MACE.	PPIs
60. Kim R B *et al*. 2023 China and Europe/USA.	Low dose aspirin for the primary prevention of cardiovascular events comparing East Asians with westerners.	Journal of American College of Cardiology. 2023	11 comparative RCTs. Meta-analysis & review.	Variable	Cardiovascular disease on prophylactic aspirin (+DAPT)	Multiple revascularization strategies	More frequent gastrointestinal bleeding in East Asians.	Cochrane *q* test & I2 statistics.	Adjusted HR/OR (95% C.I.), *P* value, other.	Aspirin + Clopidogrel/Ticagrelor/Prasugrel
								Forrest plots.		
							No significant difference in MACE rate.			
							Risk of major bleeding during aspirin vs control was greater in the East Asian population.			
66. Costa F *et al*. 2023 Multiple -international	DAPT duration after PCI in high bleeding risk: a meta-analysis of randomized trials.	European Heart Journals 2023	11 RCTs Evidence based systematic Review.	Variable Between-(from 1 month, 3-month vs 6 months standard DAPT therapy)	ACS on DAPTs including High bleeding rate (HBR) patients.	Multiple revascularization strategies.	DAPT reduced GI bleed risks among shorter 1/3 months as compared with standard DAPT (6 months therapy). No statistical difference in all-cause mortality, MACE, MI, or stent thrombosis	I2 statistics. Forrest plots.	PUD & concomitant warfarin – RF for upper & lower GI bleed.	Additional drug management
									PUD	
									Lower 74% vs Upper 26%	
68. Pelliccia F *et al* 2022 Italy	Risk Scores of bleeding complications in patients on DAPT: how to optimize identification of patients at risk of bleeding after PCI	Journal of Clinical Medicine 2022	7 RCTs analysis	Various validation cohort from 2010 to 2019.	ACS patients on DAPTs after PCI	PCI ± CABG	Serial evaluation & recalculation of bleeding risk scores during follow-to improve the identification of patients at higher risk of bleeding while on DAPT after PCI.	Sensitivity, specificity analysis, and Area under the curve.	GIB-HR: 3.40 (1.67–6.92)	PPI
										Multiple DAPT combinations.
									*P* = 0.001	
									MACE-HR: 3.75 (1.99–7.07)	
									*P* < 0.001	
74. Eisenberg *et al* 2008 Canada	Safety of short-term discontinuation of Antiplatelet therapy in patients with DES.	Circulation 2009	Modified case only design.			PCI (with angiographically confirmed DES) only.	Aspirin component of DAPT therapy: if maintained, short-term discontinuation of thienopyridine – relatively safe in patients with drug-eluting stents	Mann–Whitney U tests, chi-square/Fishers exact tests, Kaplan Meier & cumulative plot.	GIB-assoc. e.g., 30d all-cause mortality: HR – 4.87. IQR – 2.61–9.08. *P* < 0.0001. -1 yr. all-cause mortality: HR – 3.97. IQR – 2.64–5.99.	
			From-Retrospective cohort of over 7 and ½ yrs.							
			Jan 2001–July 2008							
				Variable-Maximum was 3.7 yr./patient.	ACS with DES and DAPT (Patients of Academic research consortium)				*P* < 0.0001. Higher stent thrombosis 5.8% vs 2.4%, *P* = 0.009	Heparin
										GP IIb/IIIa inhibitor-abciximab, Aspirin ± (DAPT): Ticagrelor/Clopidogrel/Prasugrel
										Ticagrelor ±prasugrel
75. Chan *et al* 2019 China	Risk of post polypectomy bleeding with uninterrupted clopidogrel therapy in an industry-independent, Double -Blind, Randomized Trial.	Gastroenterology 2019	RCT	Up to 6 months/patient.	ACS on clopidogrel ± DAPT	Multiple revascularization strategies	Higher incidence of delayed post polypectomy bleeding (up to 30 days) in clopidogrel treated group. No difference in both immediate polypectomy bleeding, & MACE at 6 months	Chi-square tests, Kaplan Meier curve estimates, Cox proportional hazard model	Adjusted HR/OR (95% C.I.), *P* value, other.	Antithrombin
										Oral anticoagulants
			Feb 2012-April 2018							GP IIb/IIIa inhibitors
										Others – include
										Aspirin, Thienopyridines, combination (DAPT).
										Antihypertensives
										Statins.
77. J Myong Lee *et al* 2012 Korea	Clinical risk factors for UGIB after PCI in a single centre study	Gut & Liver 2016	Retrospective cohort (narrative)	1 year	ACS after PCI: stent implantation (at least 1 year)	PCI only	UGIB rate after PCI = 1.1% & timely endoscopy needed	*T* tests, Mann Whitney U tests and multivariate logistic regression analysis.	GI bleed-RR East Asians: 3.29 (2.26–4.80) vs RR Westerners: 1.56; (1.29–1.88) – *P* = 0.001.	Additional drug management
									MACE rate – RR of East Asians: 0.87; (0.71–1.05)	
									RR of Westerners: 0.90 (0.85–0.95).	
									Risk of major bleeding during aspirin vs control in East Asian RR: 2.48 (1.86–3.30) compared with Westerners RR: 1.45 (1.26–1.66) – *P* = 0.001	
78. Costa F *et al* 2018 Multinational (mainly Europe and America continent)	DAPT duration based on ischemic & bleeding risk after coronary stenting.	Journal of American College of Cardiology 2019	8 RCTs meta-analysis	Variable	Severe Coronary artery disease (CAD). (22% with complex PCI)	All revascularization strategies (Including both 1st generation and 2nd generation DES stents.)	Higher rate of ischemic but not bleeding events (regardless of complex PCI features)	Cox logistic regression model.	DAPT reduced -major/clinically relevant non-major bleeding RR: 0.76 (0.61–0.94) I 2 = 28%, major bleeding RR: 0.80 (0.64–0.99), I 2 = 0%), cardiovascular mortality (RR: 0.79, 95% CI: 0.65–0.95, I 2 = 0%).	Heparin, Dabigatran,
										Warfarin
										Ticagrelor
										Various antihypertensive drugs, diuretics, digoxin & amiodarone
79. Wakabayashi Y *et al* 2014 Japan	Major adverse Cardiovascular & Bleeding events associated with non-cardiac surgery in coronary artery disease with or without prior PCI	Journal of Cardiology 2015	RCT	30 days after Non cardiac surgery	ACS patients ± PCI on low dose aspirin therapy.	PCI only	Rate of MACE not significantly different among PCI vs non-PCI patients.	Linear regression analysis	Adjusted HR/OR (95% C.I.), *P* value, other.	Aspirin.
										Antiplatelet agents.
										Anticoagulants.
										PPI Prophylaxis.
										Others.
82. W J Kikkert *et al* 2017 Netherlands	Optimal duration of DAPT for coronary artery disease	Netherlands Heart Journal 2018	11 observational and RCT studies review	Variable	Coronary artery disease ± revascularization therapy	All revascularization therapies available	Both 6 months and 1 year duration of DAPT effective.	Not clearly stated	Au-ROC of the PRECISE-DAPT score at follow-up – excellent (c-index = 0.84) for prediction of bleeding events at 1-year follow-up, vs performance of baseline PRECISE-DAPT-modest (c-index = 0.59).	Aspirin + other antiplatelet agents.
										Thrombolytic drugs excluded.
83. Costa *et al* 2018 Switzerland	Optimal duration of DAPT after coronary stent implantation	Cardiovascular Diagnostics Therapeutics 2018	18 RCTs review	Variable	Acute coronary syndrome after PCI (stents-of various types)	PCI only ± previous CABG	DAPT duration should be individualized on bleeding risk status.	Not clearly stated	Median time of Late stent thrombosis LST after PCI – 395 days.	
									Halt DAPT – median time to event – 7 days vs halted	
									Thienopyridine only – 122 days. *P* = 0.0001	Additional drug management
86. Wen Zhen *et al* 2015 China	Prediction of Gastrointestinal bleeding events in patients with ACS undergoing PCI	Medicine 2020	Prospective observational Cohort study (STROBE compliant)	Greater than 6 months duration.	Acute coronary syndrome after PCI	PCI only	Independent risk factors of bleeding (1 yr. after PCI) = History of peptic ulcers, tumor, renal & cardiac insufficiency & prolonged APTT	Multivariate Cox proportional hazards regression models	Incidence – delayed post polypectomy bleed – 3.8% clopidogrel grp vs placebo 3.6%	Not stated for each study
									HR – 1.05. (0.26–4.2). *P* = 0.946.	
									Incidence – MACE − 1.5% clopidogrel grp vs placebo 2.0%	
									HR – 0.76. (0.17–3.38). *P* = 0.714	
89. Wan J *et al*. 2016 China	Predictors and Management of antiplatelet related bleeding complications for ACS in Chinese elderly patients	Cellular Physiology & Biochemistry	Retrospective cohort study	7 years follow up.	ACS in >75 yrs. old patients after successful PCI	PCI ± CABG	Risk predictors of antiplatelet related bleeding: Female gender, BMI, Previous history of bleeding, Fasting blood sugar & chronic total occlusion.	Multivariate logistic regression analysis	Adjusted HR/OR (95% C.I.), *P* value, other.	Aspirin
										Thienopyridine – multiple and unspecified.
				Median – not clearly stated						
90. Kang *et al*. 2018 Korea	Aspirin vs Clopidogrel for long term maintenance monotherapy after PCI (HOST-EXAM Extended Study)	Circulation 2022	RCT	Median follow up – 5.8 years with Landmark analysis at 2 years.	ACS post PCI (with DES) on DAPT for 12 ± 6 months	PCI, ± CABG	Composite all cause –mortality – no significant difference	Cox proportional logistic regression analysis	Independent risk factors:	Clopidogrel among other DAPT-unspecified
									– history of PUD; OR = 12.68 (2.7–59.66), *P* = 0.001	
							MACE & BARC type 3 compared (greater bleeding)			
									– use of anticoagulants: 0 R = 7.76 (2.1–28.66), *P* = 0.002	
							Secondary events-MACE-Lower for clopidogrel group			
							Bleeding side effect-lower for clopidogrel group			
91. Quian Y *et al* 2021 China	Incidence & risk factors of aspirin therapy related bleeding complications among elderly patients after coronary stenting. A multicentre retrospective observation	Frontiers in Pharmacology 2021	Retrospective cohort study	1 year	Coronary artery disease (CAD) patients ≥ 75 yrs. old + PCI & 1 yr. DAPT	PCI, ± CABG	Incidence of BARC ≥ 2	ANOVA, Kruskal–Willis test, H test, Pearson chi-square/fishers test	MACE for Long term DAPT	Additional drug management
							Type of bleeding higher in PRECISE DAPT scored patients.		AR difference	
									Complex PCI – 3.86% (−7.71 to +0.10)	
									Noncomplex PCI – 1.14 % (−2.26 to − 0.02)	
								Au-ROC		
							Higher odds of previous stroke, MI and PUD. PCI, ± CABG			
								Univariate, logistic, and multivariate regression analysis.		
97. Harris J *et al* 2023 England	Bleeding risk in patients prescribed dual antiplatelet therapy and triple therapy after coronary interventions: the ADAPTT retrospective population-based cohort studies	Health Technology Assessment 2023	Retrospective cohort study (target RCT format)	8 years	ACS of ≥ 18 years undergoing CABG/PCI or conservatively managed ACS	CABG/PCI or none	Incidence of any bleeding – 5% among CABG, 10% among conservatively managed & 9% among PCI, compared with 18% among patients prescribed triple therapy.	Cox logistic regression model & Kaplan Meier. Logistic regression analysis	MACE events – 3.8% vs 3.9%, *P* = 0.97	Aspirin
										Other antiplatelet therapy-unspecified
										PPI-unspecified
									Bleeding events – 18.9% vs 10.4%, *P* = 0.17	
		NIHR								
97. Jian Z *et al* 2011 China	PPI can decrease gastrointestinal bleeding after PCI	Science Direct Clin Res 2013	Case control study	I year of PCI	Coronary artery disease (CAD) & ACS on DAPT	PCI only	Risk factors increase incidence	Independent 2 sample Student *T* test, logistic regression analysis	Adjusted HR/OR (95% C.I.), *P* value, other.	Aspirin, Clopidogrel, Ticagrelor, Prasugrel, PPI, ACE inhibitors, ARBs, Statins among others.
							– Advanced age, female gender, smoking disorder, PUD, previous GI bleed and PPI used to lower risk			
98. Morneau K *et al* 2011 USA	Analysis of gastrointestinal bleeding prophylaxis in patients receiving DAPT with Aspirin/clopidogrel	Journal of Management Care Specialty 2014	Retrospective review	10 months & 6 months after discharge	Cardiovascular disease on DAPT	Not clearly stated – Mainly conservative & Some revascularization therapies	Appropriate GI prophylaxis in 48%, 56.4% met guideline criteria for GI prophylaxis but did not receive PPI at discharge. 43.5% had GI prophylaxis when not needed	Logistic regression analysis	Meta-analyses unavailable	Aspirin ± Anticoagulants, Thienopyridines, Other DAPT, Unspecified drugs

ACS, acute coronary syndrome; CAD, coronary artery disease; DAPT, dual antiplatelet therapy; PCI, percutaneous coronary intervention; CABG/CAG, coronary artery bypass graft; UGIB, upper gastrointestinal bleeding; LGIB, lower gastrointestinal bleeding; PUD, peptic ulcer disease; MACE, major adverse cardiac events; BARC, bleeding academic research consortium; HBR, high bleeding risk; HTPR, high on treatment platelet reactivity; RCT, randomized control trials; *P, P* value; CI, confidence interval; PP, per protocol; ANOVA, analysis of variance; Cox proportional analysis, Cox logistic regression analysis; OR, odds ratio; HR, hazards ration; aHR, adjusted hazards ration; RR, relative risk; PPI, proton pump inhibitors; H2RA, histamine 2 receptor antagonists/blockers; GPIIB/IIIa, glycoprotein 2A/3B receptor antagonist/antibody.

### Severe acute coronary events

Myocardial infarction (MI), stent thrombosis, and major adverse cardiac events (MACE) were among the severe acute coronary events that were examined in the included studies see idiazabal *et al* gsn 43 and Costa F *et al* gsn 78. The effect of GI bleeding on ischemic outcomes, including the severity of ischemia episodes and the likelihood of death, has been the subject of many investigations. Few studies, however done in the eastern part of the world recorded the MACE, see Chan *et al* gsn 75, Wan J *et al* gsn 89, Kang *et al* gsn 90 and quan Y *et al* gsn 91. In literature however ischemic events are a major outcome of the PCI complexity as described later.

### Coronary revascularization and dual antiplatelet agents (DAPT) used in the studies

Patients with acute coronary syndrome (ACS) who underwent revascularization were traditionally managed with DAPT, which included aspirin in addition to a P2Y12 inhibitor like clopidogrel, ticagrelor, or prasugrel. Patients with CAD received different interventional therapeutic modalities, such as PCI with or without bare metal or drug eluting stents (DES) – see Huang *et al* gsn 40, Myong Lee *et al* gsn 77 vs Zhu Y *et al* gsn 36, Yasuda *et al* gsn 41 Idiazabal *et al* gsn 43, Eisenberg *et al* gsn 74 respectively. CABG only was the therapeutic modality in Sunil Rao *et al* gsn 26, Both PCI and CABG were therapeutic modalities in few eastern countries like Wan J *et al* gsn 89 and Quian Y *et al* gsn 91. Many other papers and literature specifically used medicinal therapy and included the baseline features of the research populations.

### Gastrointestinal bleeding in studies

The studies showed a wide range of prevalence rates for GI bleeding. For example, a single trial found that the rate of GI bleeding was 1.7% in the PPI group and 2.1% in the placebo group (aHR: 0.88, 95% CI: 0.46–1.66) – see Vaduganathan *et al* gsn 33. There was an increased incidence of GI bleeding in another trial, with 22% of patients not using PPIs compared to 66% of patients on PPIs (OR: 0.37, 95% CI: 0.23–0.61, *P* = 0.001) see Khan M Y *et al* gsn 35. Patients using aspirin plus clopidogrel had a 2% risk of GI bleeding in a retrospective cohort analysis; taking PPIs protected against this risk (HR: 0.10, 95% CI: 0.010.71) when compared to not taking PPIs see Huang K W *et al* gsn 40. In a different trial, patients who did not receive antisecretory medication after coronary stenting had a cumulative incidence of GI bleeding of 4.5% at 1 year and 9.2% at 2 years – see Yasuda *et al* gsn 41. Some studies provided rates that corresponded to the severity of bleeding episodes, which were often classified as mild, moderate, or severe.

### Common bleeding scoring systems available

The use of bleeding scoring systems to estimate the risk of GI bleeding in ACS patients has been discussed in much research. One such system is ARC-HBR, which stands for the Academic Research Consortium for High Bleeding Risk. Blee MACS, a multicenter registry of patients discharged with an ACS diagnosis; CREDO-Kyoto, an outcome study in Kyoto for coronary revascularization; GUSTO stands for “global utilization of streptokinase and Tissue plasminogen activator (TPA) for occluded coronary arteries.” PRECISE-DAPT predicts bleeding problems in patients following stent implantation and subsequent dual anti-platelet treatment – see Pellicani F *et al* gsn 68 & Quian Y *et al* gsn 91. Factors such as age, creatinine clearance, hemoglobin, white blood cell count (WBC), and history of spontaneous bleeding are included into the PRECISE-DAPT score. Scores of 25 or more indicate a high bleeding rate, whereas scores below 25 indicate a nonHBR statistic.

## Discussion

### The dual antiplatelet agents and cardiovascular disease?

Treatment and prevention of thromboembolic diseases, such as acute coronary events, that may occur in CVD, are aided by antiplatelet medications, which disrupt platelet activity. Aspirin and clopidogrel are the two most researched dual antiplatelet regimens. Aspirin inhibits cyclooxygenase COX 1 and 2, whereas clopidogrel inhibits P2Y12. The direct platelet effect of aspirin may be overshadowed by its adverse effects on the cardiovascular system, such as fluid overload, when taken alone. However, the class effect of P2Y12 receptor antagonists interacting with aspirin may contribute to the tolerability of DAPT in patients with cardiovascular disease^[^16^]^. Researchers from all around the world have looked at many types of antiplatelet treatment, and each DAPT has its own unique set of numerical benefits. Aspirin may be used alongside other antiplatelet medications either orally or intravenously.

Patients with unstable angina, non-ST segment raised myocardial infarction, or ACS (consisting of unstable angina and other acute coronary events) may have the most extensive history of acute coronary events. It is equally possible for angiographically identified one, two, or three vessel coronary artery pathology to be part of a patient’s extensive coronary artery history with this condition.

When treating coronary artery disease, clinicians must choose between two conflicting risks: the danger of bleeding, particularly in the GI system, and the risk of cardiovascular thrombosis^[^17^]^. By permanently inactivating a platelet protein (enzyme receptor), an ideal antiplatelet drug would take advantage of a “hit and run” strategy^[^18^]^.

### Incidence, predictors, and risk factors of GI bleeding from DAPT use on coronary artery disease

The most prevalent side effect of DAPT is GI bleeding since it directly damages the mucosa lining the digestive tract. On the flip side, there is the possibility of MACE due to medication noncompliance in patients who need long-term DAPT treatment.

In a comprehensive review comparing DAPT treatment with aspirin and clopidogrel across racial groups, the newer and more powerful antiplatelet drugs, primarily P2Y12 inhibitors such as prasugrel and Ticagrelor, did not provide overall clinically meaningful improvements three times^[^19-21^]^.

Chronologically, the frequency of UGIB for adults greater than 18 years of age with background CVD is 14.5% in a RCT involving four antiplatelet agents^[^22^]^. Aspirin adverse effects were dose dependent in terms of odds ratio, while Triflusal – another COX inhibitor, was associated with no statistically significant risk^[^22^]^.

A single center retrospective study done in Israel reported that 0.9% of patients with ACS developed UGIB in its coronary care unit over a period of 10 years^[^20^]^. Alli O *et al* recorded the rate of GI bleeding as 2.7% post revascularization therapy (mainly PCI with stenting ± CABG), after conducting an observational case control study in a single centre in the United States over a period of 4 years, which was completed in the year 2007^[^23^]^. Between the years 2011 and 2013, Huang K W and Tanigawa *et al* and Jian Z *et al* in Japan stated UGIB prevalence rates of 1–2% mainly in severe coronary artery history patients with PCI done mostly within a year^[^24-26^]^.

Significant risk factors for GI bleeding in patients on antiplatelet treatment after PCI included advanced age, a history of peptic ulcer disease, the use of non-steroidal antiinflammatory medicines (NSAIDs) concurrently, the use of combined anticoagulants, psychological distress, and other medical conditions^[^25^]^.

A recent multi-center retrospective study on elderly Chinese patients over a 10-year period objectively assessed the incidence of the Bleeding Academic Research Consortium (BARC) grade >2, among patients with ACS who had PCI and/or CABG on DAPT and found that a very high percentage of patients – 9.74% had bleeding complications^[^27^]^.

Thrombotic events are more common in Africans (or African Americans) and East Asians, while bleeding events are more common in Asians, suggesting that ethnicity is a significant factor in the occurrence of these complications after antithrombotic treatment^[^28^]^.

There are several variables that increase the likelihood of GI bleeding, including advanced age, smoking, GI disorders, and renal impairment, which is especially problematic after PCI and stent implantation. Multivariate logistic regression analysis of observational studies has demonstrated that the type of antithrombotic agent is a predictor of GI bleeding^[^29^]^, but this does not happen in isolation from other risk factors such as advanced age, critical illness requiring mechanical ventilation, or a history of peptic ulcer disease^[^24,29^]^. The most important risk factors for GI bleeding in patients with ACS after PCI were physiological stress, advanced age, and peptic ulcer disease, according to a systematic analysis that sought to identify the optimal treatment for this condition^[^25^]^. Revascularization therapy for severe coronary artery disease was associated with a high 30-day and 6-month death rate and high Global use of streptokinase and TPA for Occluded Coronary Arteries (GUSTO) scores of bleedings, according to a very large, randomized control study including 26 452 patients^[^30^]^. Patients who bled were more likely to be older and sicker, according to the study’s primary result.

The likelihood of GI bleeding episodes may be influenced by factors such as the kind of primary-lifesaving emergency coronary intervention performed, the length of multiple DAPT utilized to avoid restenosis, and the severity of acute coronary events in patients. Temporary cessation of the P2Y12 inhibitor medications in DAPT treatment therapy is only justified for elective operations in the treatment of significant acute coronary events, according to the ACG and CAG clinical practice recommendations^[^31^]^. Patients with drug eluting coronary stents are strongly encouraged to resume taking their P2Y12 receptor inhibitor as soon as possible, ideally within 5 days after endoscopy intervention, according to the APAGE and APSGE guidelines. The Task Force does not advise withholding both antiplatelet agents owing to the high risk of stent thrombosis^[^32^]^.

The incidence of symptomatic stomach damage was 7.9% in research conducted by L Zhong *et al* on 603 patients who were followed up for at least 1 year. The incidence of GI problems and symptoms seem to rise in the first 3 months after DAPT. By using COX regression analysis, the variables that increase the likelihood of severe G I problems might be discovered. In that research, the independent risk factors for significant GI problems were a history of peptic injury and a drinking habit^[^33^]^ respectively. This is consistent with research conducted in Korea, Japan, and China, among other east Asian nations, and there are little variations in the independent variables that affect the final statistical analysis^[^26,34,35^]^.

Although older age is associated with an increased risk of GI bleeding in most western studies, this finding in the L Zhong *et al* study^[^33^]^, which contradicts previous research by Huang *et al* and Watanobe H *et al*, may be due to demographic differences in the Chinese population that have not been fully investigated^[^24,36^]^. An interesting finding from a retrospective study of Chinese elderly patients (75 years and older) with ACS following successful PCI and coronary artery bypass graft (CABG) surgery was that chronic total occlusion, body mass index (BMI), and female gender were risk factors for antiplatelet-related bleeding complications (OR – 2.96, OR – 1.54, respectively)^[^37^]^. According to some research, the biggest known predictor of major GI bleeding in ACS patients is the combination of aspirin and thienopyridines, which increases bleeding in individuals with pre-existing benign or malignant GI lesions^[^34,37,38^]^.

According to research comparing the bleeding rates of different antiplatelet medicines using the venous occlusion/re-stenosis paradigm, glycoprotein IIb/IIA inhibitors had a greater risk of bleeding events compared to thienopyridines and P2Y12 inhibitors^[^20,38,39^]^. In contrast, a hazard ratio of 1.8 was related with glycoprotein IIb/IIIA inhibitors, compared to clopidogrel 2.82 in 100 patients in China who had STROBE complaints during a 6-month period. Nonetheless, nomogram-based risk assessments, risk scoring systems, and risk prediction models were developed and implemented^[^34^]^.

Looking at studies outside southeast Asia, there are several factors that contribute to the increased mortality and nonfatal MI rates in individuals with ACS who have GIB. Patients with advanced chronic kidney disease and ACS have a poorer prognosis since they have an unfavorable baseline clinical profile, which includes being older, having a greater frequency of diabetes, anemia, and chronic renal insufficiency. In the known large multicenter randomized ACUITY (Acute Catheterization and Urgent Intervention Triage Strategy) trial, Eugenia Nikolsky *et al* found that GI bleeding was more common in older patients, those with anemia, and smokers. This was a post hoc analysis using data from the trial^[^38^]^.

Patients who were older, smokers, had renal insufficiency, a history of cancer, or had experienced GI bleeding previously had greater risk of bleeding complications, according to another research^[^39^]^. Age, a history of GI bleeding, a history of cancer, smoking, and triple antithrombotic medication were the multivariate factors that predicted GI hemorrhage here.

Systematic evaluations of observational and longitudinal studies have consistently shown that PPI usage is protective for GI bleeding in people on DAPTs with cardiovascular disease^[^25,40,41^]^. Compared to histamine 2 receptor antagonists (H2RAs), proton PPIs are superior gastroprotective agents for DAPT patients. Despite no change in the reported MACE in such studies, PPIs do seem to be associated with heightened on-treatment platelet reactivity (HTPR). Present evidence suggests that PPIs may be appropriate for usage in certain DAPT patients at risk for GI complications^[^41^]^.

Multiple RCTs and retrospective studies in East Asians have examined the effects of PPIs on coronary artery disease^[^21,24^]^, and the results have been positive. However, a meta-analysis by Costa *et al* found that PPIs’ protective factors against acute GI bleeding in patients with severe ACS on DAPT are not without an increase in ischemic events^[^12^]^. While some research suggest that the presence of *Helicobacter pylori* increases the risk of GI bleeding, treatment of *H. pylori*, when identified during endoscopy, reduces this risk, there are other studies that suggest the opposite^[^42,43^]^.

### Clinical presentations and impact of gastrointestinal bleeding from DAPT use on ACS

Several aspects focusing on appropriate treatment methods, procedures may be used to study the frequency and clinical consequences of GI bleeding in individuals with ACS. Clinical correlates and treatment modalities impacted by pharmacologic drug therapy (e.g., thrombolytic therapy or DAPT) might be revised.

A meta-analysis revealed that compared to Westerners, East Asians had a much greater incidence of GI hemorrhage^[^44^]^ Despite a weaker reaction to clopidogrel, a review found that East Asians had comparable or lower rates of ischemic events after PCI than Westerners, even though the two groups had different baseline characteristics. There is a lot of regional variances in the clinical presentation and follow-up of patients with ACS on DAPT, and one reason for this already mentioned phenomenon called the East Asian Paradox^[^45^]^.

In the United States, there is a strong correlation between the severity of bleeding and an increasing risk of both short-term and long-term mortality in the research conducted by Rao S V *et al*, which found that patients with ACS often have bleeding. This bleeding may have both short-term and long-term harmful consequences, as determined by the GUSTO bleeding classification. 27% of the studies included in our meta-analysis used different terminology when describing bleeding categorization systems^[^46^]^. The Food and Drug administration (FDA) encourages the use of verified, standardized end point definitions that accurately represent clinical outcome. The FDA encouraged such initiatives after recognizing the significance of BARC’s (Bleeding Academic Research Consortium’s) worldwide strategy to a consensus set of bleeding criteria^[^47^]^. The BARC produced the most objective system for classifying bleeding severity, proposing five distinct categories of bleeding^[^19^]^. In Type 1, bleeding is not considered actionable. In Type 2, any visible symptom of hemorrhage that may be acted upon is considered actionable. In Type 3, there is evidence of bleeding from clinical, laboratory, and imaging studies. This evidence is further classified into 3a, which refers to overt bleeding with a hemoglobin decrease of 3 to <5 g/dl. 3b – seen bleeding with a hemoglobin decrease of at least 5 g/dl 3c – bleeding that causes hemorrhage within the brain or eyes, which may impair vision, Type 4 bleeding occurs within 48 hours after a coronary artery bypass graft (CABG), whereas Type 5 bleeding is fatal and may occur anywhere in the body, including the brain, lungs, genitourinary system, and retroperitoneal space^[^19,47^]^.

Swiss researchers Kosikinas *et al* looked at the causes and effects of GI bleeding after PCIs in a prospective single-center cohort study that ran from 2009 to 2012. DES were administered to the patients without restrictions. Patients were given acetylsalicylic acid (aspirin) indefinitely as part of a DAPT regimen that also included a P2Y12 inhibitor either before, during, or just after the surgery. In this setting, DES usually required a 12-month DAPT, but bare metal stents or balloon angioplasty required at least 1 month (a background research protocol)^[^39^]^.

A total of 45.5% of patients received treatment for stable coronary artery disease, 28.8% for non-ST-segment-elevation ACS, and 25.7% for STEMI. Among the patients, 64% experienced upper GI bleeding, which was more common than lower GI bleeding (36%). Additionally, the occurrence of upper GI bleeding was less common within 30 days compared to lower GI bleeding^[^39^]^. This European investigation indicated that the GI tract was the most prevalent source of bleeding 1 year after PCI, which is consistent with earlier findings from the analysis of the ACUITY trial^[^38^]^. A similar finding in individuals without coronary revascularizations and with stable coronary artery disease^[^39^]^ supports this. With the switch from femoral to radial access, the risks of major bleeding at the access site, surgical repair of the access site, transfusion of blood products, death, myocardial infarction, and stroke have been diminished^[^19^]^.

It is important to highlight the impact of GI bleeding on patients with severe ACS. In a prospective study conducted by Koskinas *et al*, there was an increased risk of all-cause mortality (adjusted hazard ratio, 3.40; 95% confidence interval, 1.67–6.92; *P* = 0.001) and the composite of death, nonfatal MI, or stroke for 1 year (adjusted hazard ratio, 3.75; 95% confidence interval, 1.99–7.07; *P* < 0.001). Similar results have been seen in other investigations^[^30,34,48,49^]^. However, when comparing PPI usage to placebo on DAPT^[^50^]^ in patients with coronary artery disease, comparative research by Bhatt D L *et al* revealed decreased upper GI bleeding hazard ratios of 0.13.

Many studies that came to comparable (observational) findings did not record important variables or confounders, which might explain why this systematic review discovered such a wide range of results. It is possible that the differing perception of the nature of therapeutic problems in comparable types of ACS patients treated with identical antithrombotic/antiplatelet medicines is due to the extended length of the ACUITY trial study. As also comprehended in the Acuity trial, patients’ inconsistent medication use outside of hospital stays, makes recall bias another factor explaining the wide range of results.

Among patients with DAPT, another prospective research in Spain revealed that lower GI bleeding was more prevalent (74% vs. 26%) throughout a mean follow-up period of 1.72 ± 1.07 years. When the bleeding occurred, 84.6% of this patient was on a PPI. This demonstrates that PPI treatment has a significant impact on the esophagus and small intestines, and the inclusion of individuals on warfarin may also contribute to the observed difference in bleeding occurrences^[^51^]^.

To the east, researchers Nagata *et al* performed a prospective single-center cohort study in Japan and discovered that compared to using NSAIDs alone (OR 2.3), the combination of NSAIDs with low-dose aspirin (OR 4.3) or other antiplatelets (OR 4.9) was significantly related with LGIB^[^52^]^.

In a famous randomized control study involving 966 centers in 52 countries, including the US, doctors can now use websites to determine the risk of bleeding for individual patients at different time periods^[^53^]^. It was the goal of this 6-year-old American experiment, TRILOGY ACS (Targeted platelets inhibition to define the ideal way to medically treat ACS), to create a longitudinal bleeding risk prediction model^[^53^]^. In this context, the Gusto and TIMI bleeding criteria are very beneficial, and solid prediction models were created for DAPT patients with ACS. A smaller study conducted in Thailand by Thanapirom *et al* in 2010 used a matched case control design to assess 981 hospitalized UGIB patients who had either a history of coronary artery disease or not. The patients were evaluated using Glasgow Blatchford scores, full rock all scores, and pre-endoscopy Rock all scores. The study accessed the Thai UGIB databases. The results for death, bleeding rates, and lengthier hospital admissions among patients with coronary artery disease were all high, however no prediction models were used. The scores were 13.2 ± 48.7 versus 4.4 ± 5.5 days^[^54^]^.

In the context of ACS, GI hemorrhage is a major complication that has been linked to mortality and ischemia sequelae, according to a post-hoc randomized open label research conducted by Nikolsky *et al*^[^38^]^ This could similarly be understood by making a reference to a RCT that recruited patients with ACS from Taiwan that used intention-to-treat analysis 2 years ago. Rates of rebleeding at 3 days were 4.55% among early endoscopy (EE) patients and 28.57% among non-EE patients, according to the rock all score used to evaluate the patients. Due to the sluggish enrollment of patients, this well-designed research was also prematurely stopped, like the ACUITY studies^[^55^]^.

Despite its rarity, GI bleeding after PCI in the hospital is a clinically relevant event associated with increased 30-day and long-term mortality^[^56^]^. It should be mentioned that intracranial bleeding (ICB) is the most severe side effect linked to DAPT. An aspirin trial for cardiovascular disease found a recurrence rate of 3.4% in deep ICBs (affecting the basal ganglia, thalamus, and brainstem) and 15.7% in lobar ICBs (affecting the cerebral cortex and underlying white matter)^[^57^]^.

An unexpected rise in MACEs was found in a recent study on bleeding risks in patients prescribed on DAPT and triple therapy following coronary interventions in the United Kingdom. This finding suggested that patients undergoing coronary artery bypass grafting who were given DAPT were at a higher risk of complications. The Aspirin Clopidogrel (AC) group was younger, more non-white, and had a greater risk of prior MI according to the baseline variables. Despite being accounted for in the statistical analysis, these factors still represent a population where secondary events like cerebrovascular accidents are more likely to occur due to the assumed growing complexity of the illness^[^58^]^.

The advantage of cangrelor, an intravenous thienopyridine derived antiplatelet medication, as regards major ischemic endpoints was offset by an increase in minor bleeding events, according to a meta-analysis of studies comparing various antiplatelet medicines. Trials like “CHAMPION PCI” (Cangrelor versus Standard Therapy to Achieve Optimal Management of Platelet Inhibition) and “CHAMPION PLATFORM” (A Clinical Trial Comparing Cangrelor to Clopidogrel Standard Therapy in Subjects Who Require Percutaneous Coronary Intervention) were involved^[^59^]^.

Bleeding unrelated to cardiac procedures that is caused by the antiplatelet medication used has been documented in systematic reviews by Eisenberg *et al*^[^60^]^ and Chan *et al*^[^61^]^. A double-blind RCT was performed by Chan *et al* to assess the rates of bleeding and thrombosis in individuals who were given either 75 mg of clopidogrel or a placebo for 7 days before to colonoscopy. A significant portion of these patients underwent cold snare polypectomy. There was a little tendency toward fewer cardiac thromboembolic events with thienopyridine discontinuation, and the rates of immediate and delayed post-polypectomy bleeding were comparable^[^60^]^. Time to late stent thrombosis (30 days to 1 year following stent implantation) in patients with drug-eluting stents on DAPT after stopping either thienopyridine alone or both thienopyridines was examined in the Eisenberg *et al* research^[^60,61^]^

The thought of ceasing antiplatelet drugs in severe ACS patients should be approached with care due to the higher risk of late stent thrombosis compared to acute stent thrombosis, as well as the potential for ischemic rather than GI bleeding.

### Management of GI bleeding in the setting of severe ACS patients on DAPT

The initial clinical presentation is the primary determinant in the therapeutic strategy for GI bleeding in patients on DAPT. When there is bleeding in the upper GI tract, it is crucial to treat the patient quickly using a combination of tight blood transfusion strategy, an emergency resuscitative procedure, and upper GI endoscopy. This is especially true in cases of large bleeds where there is platelet activation and creation of prothrombotic milieu^[^19^]^.

Since PCI is a class 1 therapeutic option for patients with ACS and is often done in patients with chronic coronary syndromes (CCS), it is usual for patients with severe ACS on DAPT to already have a PCI set up when they visit^[^62^]^. If a patient has 1- or 2-vessel disease, including the proximal left anterior descending artery (LAD), most facilities considered PCI as observed in our review. On the other hand, coronary angioplasty (CABG) is considered for patients with 3-vessel or left main stem disease, and on rare occasions for patients with 1- or 2-vessel disease, according to current guidelines.

Using information from 2014, Bilal *et al* examined the Nationwide Readmission Database. The data included here originate from more than 2000 institutions and include about 15 million hospitalizations, making it the biggest readmission database in the United States. Everyone who had ACS and a stent placed during the initial hospitalization made the cut: 22 660 individuals. Among the patient characteristics, 74% had a history of drug-eluting stent implantation^[^63^]^.

The American College of Cardiology (ACC) recommendations state that, except in life-threatening emergencies, ED risk assessments are necessary to screen out patients with very low risk profiles (e.g., Glasgow–Blatchford score: 0–1 for Upper GI bleed, & Oakland score of 8 points or lower for lower GI bleed), who might be released with outpatient follow-up. The presence of the comorbidity: background coronary artery disease history increases such scores mentioned above and majority of these patients are hospitalized. Patients admitted to the hospital with hemorrhage from the esophagus or stomach should get a transfusion of red blood cells at a level of 7 g/dL^[^19^]^. Infusion of erythromycin prior to endoscopy and endoscopy no later than 24 hours after presentation^[^64-66^]^. Continuous or intermittent high-dose PPI treatment for 3 days is indicated following endoscopic hemostasis^[^66^]^, followed by twice-daily oral PPI for the first 2 weeks of therapy after endoscopy. When endoscopic treatment fails to stop recurrent bleeding, transcatheter embolization becomes the next recommended course of action^[^67^]^.

Resumption of ASA within 24 hours following successful endoscopic hemostasis should be explored if the drug is stopped at the clinical presentation of bleeding in patients with severe ACS on DAPT treatment. 15, however, an observational analysis found that all patients with cardiovascular disease were more likely to rebleed and less likely to have ischemic events or death if they resumed anticoagulant or antiplatelet medication. This was true for both UGIB and LGIB^[^68^]^.

Cardiology expert consults are required for blood transfusions in actively bleeding ACS patients on DAPTS and for target HB required before invasive treatments such as endoscopic hemostasis. According to Saltzman *et al*, cardiologist consultations should be part of the management of acute GI bleeding before a P2Y12 inhibitor is withheld. If a P2Y12 inhibitor is withheld, resuming treatment quickly after bleeding control can decrease the risk of MACE^[^69^]^.

Several risk scoring systems can be utilized to assess the likelihood of bleeding complications following a procedure and to determine whether a patient with stable coronary artery disease who is on DAPTS and not experiencing active bleeding is ready for non-cardiac surgery. While there was no statistically significant difference in the incidence of bleeding between the PCI and no PCI groups, Y. Wakabayashi *et al* found that the risk of MACE after non cardiac surgery (NCS) was greater in the PCI group. Despite severe coronary artery stenosis, the research found that individuals with stable CAD might potentially undergo NCS without revascularization^[^70,71^]^.

Evaluating patients with ACS on DAPTS and monitoring for bleeding tendencies is now common practice, as is developing risk prediction models and risk ratings. When it comes to monitoring patients with coronary artery disease who are on antithrombotic medication and evaluating bleeding episodes, the PRECISE DAPT has shown promising clinical outcomes with its encouraging Area under the curve – Au ROC^[^3,4,53,72^]^. Research from the RE-SCORE registry found that although the PRECISE-DAPT score’s efficacy increased with frequent reassessment, its external validation at baseline was only adequate^[^3,72^]^. According to these results, the risk of bleeding is dynamic and the only way for the score to work in practice is to recalculate it at each follow-up. It is possible that these ratings might provide light on the issues faced by unclear occurrences in previous trials and lead to warnings in the clinical profiles of many individuals. Patients with high-risk features should have a comprehensive evaluation of the risk of GI bleeding during PCI, according to several studies’ clear recommendations for the administration of triple antithrombotic treatment^[^22-25,27-33,37-39,73^]^.

To address the “East Asian Paradox,” concepts such as a “race tailored antithrombotic treatment” for CAD and PCI patients are being considered. This approach would use prediction scores that are specific to the eastern world’s youth and old age groups^[^74^]^. Age, history of spontaneous bleeding, and creatinine clearance are three factors that might change over time and affect the PRECISE-DAPT score. The latter two may decline after a PCI due to the increasing worsening of renal function caused by acute kidney injury^[^75^]^.

The ideal length of DAPT treatment influences the subsequent care of patients, which in turn relies on the kind of DAPT therapy performed. Recent acute coronary events of 4–6 weeks, stopping DAPT in DES in 1 year, and stopping bare metal stents in 1 month are all examples of recent situations that increase the risk of thromboembolic events.

Similar to the APAGE and APSDE recommendations, the BSG-ESGE guidelines address the suspension and resume of antiplatelet and anticoagulant medication during endoscopic operations. After endoscopic hemostasis, it is highly advised to resume aspirin use as soon as possible, ideally within 3–5 days^[^76^]^.

It is highly discouraged to withhold both antiplatelet medicines from patients on DAPT who have coronary stents, since this greatly increases the risk of stent thrombosis. The Task Force’s recommendation was to keep patients taking aspirin while removing clopidogrel from those on PPI infusion and DAPT. It is highly advised that patients who have drug-eluting coronary stents resume taking their P2Y12 receptor inhibitor as soon as possible, ideally no later than 5 days after endoscopic hemostasis^[^32^]^.

The extent to which platelets are inhibited during therapy dictates how efficiently platelets recover upon discontinuation of P2Y12. After 7 days of using prasugrel, 5 days of taking clopidogrel, and 4–5 days of taking ticagrelor, platelet reactivity usually returns to baseline by washout^[^77^]^. It seems that aspirin may not be very effective in cases of severe P2Y12 blockage, as L traby *et al* found that ticagrelor monotherapy and ticagrelor-based DAPT had comparable effects on the activation of the hemostatic system^[^78^]^.

The median time from PCI to late stent thrombosis (LST) was longer in patients with sirolimus-eluting stents compared to Paclitaxel, according to a prior systematic review study conducted by Canadian researchers Eisenberg *et al* on the safety of short-term discontinuation of antiplatelet therapy in patients with DES (first generation). The research conducted by Feres F *et al*^[^60^]^ used Zatarolimus eluting stents and found that short-term DAPT (3 months) was comparable to long-term (12 months) DAPT in terms of mortality, MI, and stroke incidence, while not substantially raising the risk of stent thrombosis^[^79^]^.

The recommended length of DAPT may vary from patient to patient based on factors such as stent type, procedural details, patient demographics, and ACS status^[^71^]^. The hypothesis that patients with a history of prior ACS may further minimize ischemic events with longer duration and more vigorous DAPT has also been underwhelming in nonstent centered studies.

Based on the ESC guidelines for the management of ACS and the available scientific evidence, there are alternatives to the standard 12-month DAPT duration for patients with ACS. One option is to shorten the duration to 1–3–6 months, depending on the balance between bleeding and ischemic risks. Another option is to de-escalate DAPT from prasugrel/ticagrelor-based to clopidogrel-based^[^80^]^.

In terms of decrease MACE among noncomplex PCI procedures (1.14%, 95% C.I. −2.26 to 0.02) and complex PCI procedures (3.84%, 95% C.I. −7.71 to +0.10) on ACS patients, the meta-analysis of eight RCTs on PCI patients living in Europe and America could be influenced by using bare metal stents, first- or second-generation DES^[^81^]^. PCI complexity relates to high bleeding rates among patients treated with DES in China, Complex PCI is usually PCI with 1 or more stents implanted, 1 or more lesions treated, and/or 3 coronary vessels, total stent length >60 mm, total lesion length >30 mm, and/or treatment of a chronic total occlusion, and the presence of at least one vessel.

In patients with severe ACS who are actively bleeding, reversal therapies to antithrombotic (anticoagulant/antiplatelet) therapy play little to no role, and patients on antiplatelet agents who are hospitalized or under observation with acute GI bleeding who are not thrombocytopenic should not receive platelet transfusions^[^21,32^]^.

Additionally, it is recommended that patients on DAPT (which includes P2Y12 inhibitors like clopidogrel, prasugrel, or ticagrelor and ASA 81–325 mg/d) for secondary cardiovascular prevention temporarily stop taking the P2Y12 inhibitor before elective procedures, but this recommendation does not apply to emergency procedures^[^31^]^.

**Figure 2.**
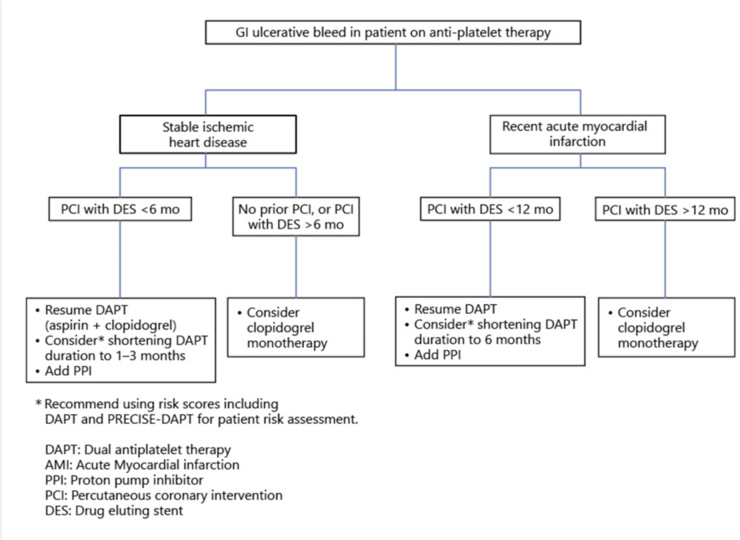
Gastrointestinal (GI) bleed in a patient on antiplatelet therapy. *Recommend using risk scores DAPT and PRECISE DAPT for patient risk assessment. Reproduced with permission from reference^[^92^]^. Copyright © 2021, Silverchair Publisher

Interventional cardiologists from many European centers who are interested in studying the ideal duration of DAPT, worked on both PCI naïve individuals and patients with stented hearts. The last 10 years have seen a proliferation of meta-analyses and systematic reviews of randomized controlled trials^[^12,42,81-87^]^. Fig. 2 is suggested as the concern for PPI clopidogrel or PPI aspirin interractions have not been a main problem to consider in previous studies which has been reproduced with permission from reference^[^88^]^. Copyright © 2021, Silverchair Publisher.

With the use of normograms, for bleeding risk calculation, Fig. 2 above is suggested above as the concern for PPI-clopidogrel or PPI aspirin interactions have not been a main problem to consider in previous studies. With recent advances in DES technology, monotherapy with a P2Y12 inhibitor, and an increase in the frequency of high bleeding rates (HBR) in eastern countries, notably Japan, a reduction in the recommended period of DAPT is presently upheld^[^87^]^. The Japanese Circulation Society (JCS) recommendations 2020 focused update recommends DAPT with prasugrel 3.75 mg daily or clopidogrel 75 mg daily with aspirin 81 162 mg daily for patients with stable CAD for at least 1–3 months after coronary stent implantation. For individuals with HBR, it may be prudent to pursue a very brief DAPT of 13 months. After 1–3 months of DAPT, patients with high thrombotic and HBR levels should be evaluated for monotherapy with a P2Y12 receptor inhibitor in accordance with the same JCS recommendation. It is suggested that patients with ACS take aspirin 81–162 mg daily in addition to prasugrel 3.75 mg or clopidogrel 75 mg for 3–12 months after coronary stent installation. Recommendation: DAPT should be shortened in patients with HBR to 1–3 months. Following short-term DAPT, individuals with high thrombotic and HBR levels should be evaluated for monotherapy with a P2Y12 receptor inhibitor, according to the JSC recommendations^[^86^]^.

All patients receiving primary PCI should take aspirin immediately, barring any contraindications, according to Saudi Arabian guidelines for the treatment of ACS. Aspirin loading doses of 150–300 mg taken orally are advised. This is prescribed before or at the latest at the time of PCI and continued for 12 months, unless there are contraindications, such as an excessive risk of bleeding, either ticagrelor (180 mg loading dose orally, followed by 90 mg twice daily for 12 months) or clopidogrel (600 mg loading dose orally, followed by 75 mg once daily for 12 months). The paper did not provide any specific bleeding risk prediction ratings, especially for dual antiplatelet drugs,^[^88^]^ However, clopidogrel should be used with aspirin for individuals undergoing fibrinolysis. For patients who are 75 years old or older, the loading dosage of clopidogrel is 75 mg, followed by 75 mg once day for 12 months. The first oral dose is 300 mg^[^89^]^.

Given that Peptic ulcer disease (PUD) is more common in patients taking aspirin or other NSAIDS, the most recent Maastricht VI/Florence consensus report from 2022 highlighted the need to test for and treat *H. pylori* in high-risk patients already taking long-term aspirin, as well as to test for and treat naïve patients beginning long-term NSAID therapy. Some people may need more than one PPI medication^[^90^]^.

### Prevention of GI bleeding in ACS patients on DAPT

Risk assessment of bleeding avoidance strategies primary aims at identifying cases with high bleeding risk. Preventing bleeding risk (not only GI) are operative on the level of de-intensifying antiplatelet therapy without compromising its potential to protect against ischemic events. For patients treated with DAPT in the early stages of an ACS, the ACCF/ACG/AHA consensus paper^[^91^]^ still suggested the use of PPIs.

Due to the continuous nature of many contentious problems, the whole prophylactic requirement of PPI on chronic coronary syndrome has not been fully assessed. It must however be known that in patients with ACS on DAPTs and already GI bleeding has occurred, there are partially overlapping different set of risk factors^[^19,92^]^. This later can be characterized as low on treatment platelet reactivity, and generally patients characteristics (age, gender, ethnicity)^[^92^]^. Importantly, most of these markers are not only markers of high bleeding risks but also that of higher ischemic risk that may explain the moderate efficacy of risk assessment tool based on therapeutic interventions^[^92,93^]^.

Despite the increased and sustained use of more effective antiplatelet and antithrombotic medicines, the incidence of GIB following PCI have fallen throughout the last decade. Modern practice, in contrast to previous studies, includes preventative therapy with PPIs and the use of antithrombotic pharmaceutical dosages that are risk-tailored, meaning they consider factors such as age, weight, and renal function^[^94^]^. This review found that several studies, including the post-hoc analysis of the ACUITY trials by Nikolsky *et al*, lacked sufficient information about the preventive therapy of PPIs for CAD patients on DAPT. PPI therapy was not included in the research. Despite randomized controlled trials^[^73,74^]^ confirming their effectiveness for secondary upper GIB prevention, several recent research have evaluated the safety and effectiveness of antisecretory medication for primary prevention of peptic ulcer formation and subsequent UGIB^[^20,21,24-33,40,41,51^]^.

Similar to the Jiang Z *et al* research, the Li Zhong *et al* prospective cohort study^[^33^]^ found that PPIs medication effectively prevented major GI problems in ACS patients after correcting for age, drinking, smoking, and prior peptic usage using Cox regression analysis^[^26,33^]^.

Previous Japanese research on UGIB in patients undergoing DAPTs after coronary stenting found a significant cumulative incidence of GI bleeding (4.5 percent with PPIs and 9.2 percent without), with higher coronary artery stenosis in the PPI group^[^75^]^. Several additional studies have failed to find any evidence that PPIs improve patients’ clinical outcomes^[^87-91,94-100^]^. The study by Al Mufleh *et al* conducted a comprehensive review comparing the use of H2 receptor antagonists (H2RAs) and PPIs in patients on DAPTs. The researchers discovered that the PPI group had a greater risk of the laboratory outcome metric HTPR, with an odds ratio of 1.28 (95% CI 1.03–1.60)^[^41^]^. When it comes to the preventative therapy of patients with atrial fibrillation on DAPT and long-term follow-up, PPIs are not without their drawbacks^[^41,101^]^.

In addition to avoiding the early end of DAPT due to rebleeding after upper GIB stabilization, PPIs have other useful effects^[^102^]^. In one study, H2RAs led to an increased incidence of mucosal erosion but not ulcer formation in individuals using low-dose aspirin for secondary prevention of cardiovascular disease^[^103^]^. For the prevention of aspirin-induced ulcer recurrence, a randomized phase 3 study also shown that PPIs are just as effective as the new potassium-competitive acid blocker Vonopraxan (TAK-438)^[^101,104^]^.

While the studies conducted by Nikolsky *et al* and Li Zhong *et al* did not assess how *H. pylori* infection and its elimination affect the clinical outcome of patients with erosive gastric or duodenal disease who are on DAPT for ACS, there have been a handful of recent studies that raise the possibility that this could impact the prognosis of GI bleeding in these patients, particularly those on long-term antithrombotic medication albeit this was a single-center study^[^43^]^.

Regarding the possible metabolic interactions between PPIs and DAPT, It has previously been proposed that a lipid-aspirin combination, as an example of a single antiplatelet treatment (SAPT), may lessen endoscopic GI ulcers when compared to immediate-release aspirin^[^88^]^. Another counterargument is that monotherapy with P2Y12 inhibitors is usually the best course of action in the time following moderate or severe GI bleeding episodes. This is because it prevents the gastric mucosa from being ulcerated by aspirin, which could lead to a recurrence of the bleeding^[^105^]^. Although there is some evidence that PPIs may reduce the inhibitory impact of clopidogrel on platelet P2Y12 receptors and increase the risk of adverse events after an ACS^[^106,107^]^. Supporters of this principal point to the fact that drug–drug interactions are likely to occur when thienopyridines, which are prodrugs, are converted into active forms through intricate biochemical reactions involving multiple cytochrome P450 (CYP) isoforms, one of which is CYP2C19, an enzyme that is also involved in the metabolism of PPIs^[^16^]^.

Contrary to the previous concept, there was no evident cardiovascular interaction between clopidogrel and omeprazole in the COGENT study, which compared the two medications in patients with coronary artery disease who were also taking aspirin and clopidogrel. Even in individuals who are not at very high risk of GI bleeding, COGENT did discover that prophylactic PPI administration does minimize the incidence of GI hemorrhage with DAPT^[^50^]^.

It should be noted that CYP2C19 has no effect on the platelet inhibitory action of prasugrel. A study found that the higher platelet inhibitory effect obtained by doubling the clopidogrel dose was completely neutralized by the coadministration of lansoprazole, but no such drug interaction was observed with prasugrel. Therefore, a clinician may want to consider switching to prasugrel instead^[^108^]^. For patients with unstable angina or MI without ST segment elevation, the Targeted Platelet Inhibition to Clarify the Optimal Strategy to medically manage ACSs trial^[^109^]^ found that prasugrel was better than clopidogrel in the subgroup of patients who used PPIs.

While taking ticagrelor and prasugrel at the same time may help prevent UGIB in DAPT patients, there are no known adverse interactions between the two drugs, and CYP2C is not involved in ticagrelor metabolism; instead, clearance is mainly through CYP3A4^[^42^]^. It is also desirable to combine PPIs with less CYP2C19 inhibition with those with higher inhibition, such omeprazole^[^94^]^. On the overall note, although there is a lack of data to support the claim that SAPT is a good antiplatelet agent, several recommendations recommend that it can be maintained in other patients^[^110^]^.

More research is needed to establish a connection between the risk factors for hemorrhagic complications seen in East Asian patients and those from other parts of the globe. It is crucial to do research on the root causes of bleeding symptoms among East Asians in order to better understand their unique clinical presentations.

The ESC recommendations have suggested using Glycoprotein IIb/IIIa receptor inhibitors only for bail out or periprocedural problems, but there was a lack of evidence on this topic in this review^[^80^]^.

## Conclusions

Correlating high risk factors of hemorrhagic complications were seen in East Asian patients compared to patients from other regions of the world. Moreover, the causes of bleeding manifestations in particular clinical conditions, among East Asians were also seen.

In the attempt to raise the bar for the treatment of cardiovascular disease, particularly in patients with an extensive coronary artery disease history, DAPT has progressed beyond the conventional dosage of clopidogrel and aspirin across the world. The use of such DAPT in managing coronary artery disease particularly post-PCI cannot be overemphasized.

The use of dual antiplatelet medications-DAPT has identified PCI with DES as the most common treatment worldwide and this has greatly improved the results of PCIs for patients with coronary artery disease. However, bleeding is a major concern since it is the most common noncardiac complication following a PCI and has a negative prognostic impact like the ischemic events, particularly major adverse cardiac events (MACEs) such as restenosis. In terms of these kinds of danger, the western and eastern halves of the globe are very different. The key findings in our review included the identification of independent predictors of GI bleeding and the frequent use of DAPT score for bleeding risk prediction.

There is need to thoroughly evaluate the risk-benefit ratio of DAPT in patients with ACS as highlighted by the increased risk of GI bleeding in Southeast Asia. Older scores are being gradually superseded by more recent clinical prediction tools, such as PRECISE DAPT and POST DAPT, both of which have shown promising clinical outcomes when following up on DAPT with ACS patients. Patients on DAPT who were at increased risk of bleeding problems should be better managed with the use of the scoring systems.

As regards antiplatelet therapy, new recommendations advise shorter optimum medication durations after revascularization treatments, and this recommendation stands even in Southeast Asia, where the problem is more severe. Research is continuing to determine whether individuals receiving strong DAPT treatment benefit from routinely taking PPIs, even if existing recommendations do not advise doing so for patients with a low risk of GI bleeding.

The topic of whether there is an ideal antiplatelet approach after GI hemorrhage in individuals with coronary artery disease has not completely been addressed with the availability of guidelines most notably in southeast Asia where there is scarce literature on the use of SAPT in coronary artery disease patients at high risk of gastrointestinal rebleeding.

## Data Availability

Available upon reasonable request.

## Research registration unique identifying number (UIN)

Registered in Research Registry having UIN reviewregistry1878.
